# Biological effects and epidemiological consequences of arsenic exposure, and reagents that can ameliorate arsenic damage *in vivo*

**DOI:** 10.18632/oncotarget.17745

**Published:** 2017-05-10

**Authors:** Chinthalapally V. Rao, Sanya Pal, Altaf Mohammed, Mudassir Farooqui, Mark P. Doescher, Adam S. Asch, Hiroshi Y. Yamada

**Affiliations:** ^1^ Center for Cancer Prevention and Drug Development, Department of Medicine, Hematology/Oncology Section, University of Oklahoma Health Sciences Center (OUHSC), Oklahoma City, OK, USA; ^2^ Stephenson Cancer Center and Department of Family and Preventive Medicine, University of Oklahoma Health Sciences Center (OUHSC), Oklahoma City, OK, USA; ^3^ Stephenson Cancer Center, Department of Medicine, Hematology/Oncology Section, University of Oklahoma Health Sciences Center (OUHSC), Oklahoma City, OK, USA

**Keywords:** arsenic, reactive oxygen species (ROS), genomic instability, antioxidants, cancer prevention

## Abstract

Through contaminated diet, water, and other forms of environmental exposure, arsenic affects human health. There are many U.S. and worldwide “hot spots” where the arsenic level in public water exceeds the maximum exposure limit. The biological effects of chronic arsenic exposure include generation of reactive oxygen species (ROS), leading to oxidative stress and DNA damage, epigenetic DNA modification, induction of genomic instability, and inflammation and immunomodulation, all of which can initiate carcinogenesis. High arsenic exposure is epidemiologically associated with skin, lung, bladder, liver, kidney and pancreatic cancer, and cardiovascular, neuronal, and other diseases. This review briefly summarizes the biological effects of arsenic exposure and epidemiological cancer studies worldwide, and provides an overview for emerging rodent-based studies of reagents that can ameliorate the effects of arsenic exposure *in vivo*. These reagents may be translated to human populations for disease prevention. We propose the importance of developing a biomarker-based precision prevention approach for the health issues associated with arsenic exposure that affects millions of people worldwide.

## INTRODUCTION

Arsenic is a poisonous heavy metal that is plentiful in the crust of the earth. It can take a variety of forms: organic and inorganic, with various valences, i.e., (III) or (V). This metal can affect human health through contaminated diet (grains, meats, and especially seafood), drinking water, and other forms of environmental exposure. There are many arsenic exposure “hot spots”, including Chile, Argentina, Bangladesh, West Bengal (India), China, Vietnam, Thailand, Taiwan, Inner Mongolia, Nepal, and Mexico. Some regions in the U.S., including California, Nevada, Massachusetts, North Dakota, and central Oklahoma, also harbor “hot spots” for ground or surface water contamination with arsenic [1–3]. As of 2016, the U.S. Environmental Protection Agency (EPA) and the World Health Organization (WHO) set the Maximum Contaminant Level for arsenic in drinking water as 10 ppb (parts per billion; 10 ppb = 10 μg/Liter). Average drinking water in the U.S. contains 2 ppb of arsenic. However, water from some regions have levels exceeding 20 ppb; e.g., 12% of water from surface water sources in the north central region, and 12% of water from groundwater sources in the western region [2]. Removing arsenic from contaminated water requires specific equipment. Activated carbon-based filters for water pitchers are commonly available in the U.S. and can remove arsenic, but in many cases do so at a poor efficiency (12–55%). Reverse osmosis can remove arsenic more efficiently, at a higher initial investment for the equipment, which may not be readily available. Various other filtration and impurity removal methods are under development; implementation will take more time. Arsenic exposure will remain a health hazard for millions of people in the near future [3].

### Effects of arsenic exposure: how arsenic could cause cancers

In case of acute poisoning, arsenic can inhibit up to 200 enzymes. The enzymes include those involved in critical cellular functions such as DNA synthesis and repair and cellular energy pathways. Symptoms of systemic acute poisoning include abdominal pain, nausea, vomiting, severe diarrhea, encephalopathy, and/or peripheral neuropathy. A lethal dose can lead to multiorgan systems failure, coma, and death. The acute minimal lethal dose is 100 to 300 mg in human adults [4].

Chronic exposure to lower-dose arsenic is associated with various symptoms. Respiratory disease, anemia, peripheral neuropathy, and peripheral vascular disorders have been reported. Hyperpigmentation and hyperkeratosis in the skin of the hands and feet are also characteristic symptoms. As epidemiological studies suggest, various cancers may develop after years of arsenic exposure [4, 5]. How exactly sub-acute poisoning doses of arsenic exposure, in environmentally relevant doses (100-several hundred ppb), increase carcinogenesis, heart disease, neuronal damage, or other disease conditions in humans remains unclear. Studies using rodent models, however, have demonstrated multifaceted effects of arsenic that include generation of reactive oxygen species (ROS), (which causes oxidative stress and DNA damage), epigenetic DNA modification, induction of genomic instability, and inflammation and immunomodulation.

### ROS generation

In rodent models, environmental-exposure-relevant doses of 50–500 ppb arsenic in drinking water for as little as 30 days led to increased ROS, gene expression changes in lymphocytes, metabolic modulation, and increased DNA damage [6–12]. A recent meta-analysis of 58 rodent studies [13] indicated that short- (>1 months) and long-term (<3 months) exposure to both low and high doses of arsenic increased systemic oxidative stress and ROS; these findings are supported by various ROS metrics, including glutathione disulfide (GSSG), malondialdehyde (MDA), and superoxide dismutase (SOD). Consistently, humans in China chronically exposed to arsenic in drinking water indicated signs of oxidation (i.e., increases in serum lipid peroxides and decreases in non-protein sulfhydryls) [14]. Thus, increasing ROS in the body is a major effect of low and high doses of arsenic exposure *in vivo*.

### Epigenetic DNA modification

Arsenic also induces genome-wide hypomethylation that can lead to genomic instability [15]. Higher levels of gene expressions are often associated with promoter hypomethylation, while lower levels of gene expression are related to promoter hypermethylation [16]. Thus, arsenic-induced hypomethylation can affect the global transcriptome.

### Arsenic and genomic instability

Another link between arsenic and carcinogenesis is induction of genomic instability. Arsenic is a strong clastogen, although it is not a classical point mutagen. Various chromosomal aberrations and aneuploidy were reported among human populations exposed to arsenic [17]. ROS generated by Arsenic and its metabolites can increase DNA damage and mutations through oxidative DNA adduct formation, DNA-protein crosslinks, and single- or double-stranded DNA breaks. In addition, they can induce telomere dysfunction, irreversible DNA impairment, mitotic arrest, and apoptosis. The aforementioned epigenetic regulation (i.e., DNA methylation, miRNA expression) also alters gene expressions. These can trigger genomic instability through Microsatellite Instability and/or Chromosome Instability [17]. As genomic instability itself is a mutator, genomic instability can lead to further mutation and genomic instability, and accelerate mutational accumulation and carcinogenesis [18]. On the other hand, excessive genomic instability can lead to cell death and tumor suppression [19], which may be a basis for using arsenic as a chemotherapy agent.

Arsenic is known to affect genes regulating genomic stability. For example, arsenic inhibits transcription of the hTERT gene (the reverse transcriptase subunit of human telomerase), possibly through decreases in c-Myc and Sp1 [20]. Arsenic induced mot-2, a p53 inhibitor [21]; thus, p53-mediated genomic integrity maintenance can be inhibited. In addition, arsenic can inhibit the DNA repair function of the Breast Cancer (BRCA) and Fanconi Anemia (FA) pathway [22]. Because arsenic can increase genomic instability through multiple pathways, a combination of interventions, instead of a single biological pathway-targeted approach, may be needed.

### Arsenic and inflammation

Inflammation is a well-known hallmark event for cancer progression, and arsenic is linked to the occurrence of cancer in highly exposed populations. Inflammation plays a key role in protecting the damaged tissues after injury, but also leads to several diseases when prolonged. Chronic sustained and persistent inflammation leads to a variety of inflammatory disorders, including pancreatitis, inflammatory bowel syndrome, and several cancers, like colon, pancreatic, liver, lung, and bladder cancer. Arsenic and arsenic-containing agents are reported to induce inflammation. For example, in Caco-2, intestinal, and HBE (human bronchial epithelial) cells, arsenic-containing reagents induced proinflammatory cytokines (e.g., TNF-α) and interleukins (e.g., IL-6, IL-8, and IL-1βn) [23, 24]. Although lower doses of arsenic might not produce characteristic skin disorders, chronic exposure to arsenic inflicts systemic inflammation, including lung inflammation, even in non-smokers [25, 26].

In a 2015 study, Dutta et al. showed that inflammatory responses and DNA damage are induced by chronic low-level arsenic exposure [27]. Women from rural West Bengal, who were chronically exposed to low-level arsenic, showed pronounced expression of CD14 on monocytes. CD14 activates downstream pro-inflammatory signaling (e.g., TNF-α, NF-κB) [27]. In laboratory preclinical studies, arsenic exposure made C57BL/6 mice prone to respiratory infections and inflammation, and caused alterations of metabolic pathways in fatty livers through inflammation [26, 28]. Arsenic also increases ROS/oxidative stress and immune dysfunction [29].

Pancreatitis is the inflammation of pancreas, characterized by pain and fibrosis that can lead to pancreatic cancer under chronic conditions. As acute pancreatitis-associated processes such as necrosis, apoptosis, inflammation, or duct obstruction can destroy the secretory parenchyma, chronic pancreatitis may develop as a result of acute pancreatitis. In 2013, about 17 million cases of pancreatitis occurred worldwide. These cases resulted in 123,000 deaths, an increase from the 83,000 deaths in 1990. Often, chronic pancreatitis starts between the ages of 30 and 40. Arsenic poisoning is one of the causes of pancreatitis. Experimental studies showed that the exposure of rabbits to arsenic trioxide resulted in elevated serum amylase activity, nitrite accumulation, and diabetes development. These findings indicate that pancreatic damage may have occurred [30, 31]. A female acute pancreatitis patient, who presented with nausea, vomiting, and diarrhea after use of an herbal tea bag that consisted of high level of arsenic, showed high urinary levels of arsenic (9000 μg/24 hours) [32].

Arsenic used chemotherapeutically (see later section) may cause acute pancreatitis, as reported by some patients. A 77-year-old patient treated for acute promyelocytic leukemia with arsenic trioxide suffered from acute pancreatitis [33], while another 26-year-old patient developed pancreatitis and toxic hepatitis after oral consumption of arsenic trioxide [34]. Although pancreatitis is not commonly associated with chemotherapeutic doses of arsenic treatment, consideration should be given for withdrawal if pancreatitis symptoms are observed.

The above literature clearly shows that continuous exposure to arsenic leads to several disorders, including inflammatory disorders and numerous cancers. Few in-depth and systematic studies have been performed to determine if arsenic leads to cancers through inflammatory diseases. It is unclear whether arsenic alone first alters inflammatory signaling, and then initiates chronic diseases. Extensive laboratory studies involving preclinical animal studies (both *in vitro* and *in vivo)* are warranted to elucidate the direct effects of arsenic on inflammation and cancer. To determine the role of arsenic in physiological and genetic alterations, effects of arsenic on gene and transcriptome need to be studied. One important approach is to use anti-inflammatory agents to counteract the effects of arsenic. Further, to determine the effects of arsenicals on humans who are continuously exposed to anti-inflammatory agents like NSAIDs, including aspirin, meta-analytic studies are needed. Recent studies have shown that new anti-inflammatory agents with dual COX-LOX inhibitory effects, like licofelone, effectively inhibit inflammatory pancreatitis and pancreatic cancer in a genetically engineered mouse model [35]. Further, licofelone's effects against arsenic, inflammation, and cancer should be studied, as ample evidence from human studies indicates that arsenic exposure enhances cancer risk.

### Arsenic and immunomodulation

In addition to inflammation, arsenic exposure can modulate immune functions by affecting cytokine levels (e.g., IL1B, IL-2, IL6, CCL2, CD14), CD4+/CD8+ T cell ratios, and the expression of immune-response genes (e.g., TNF, IL11, IL10RB, CCR1, and CXCL2) [36]. Fetal immune system can be altered by *in utero* exposure to relatively low levels of arsenic, which may lead to immune dysregulation. In a study in New Hampshire, US, a human pregnant cohort exposed to environmental arsenic from well water was examined. Absolute total cell counts for CD45RA+ CD4+ CD69+ cord blood T cells were lower with higher maternal urinary arsenic concentrations (*N* = 116, *p* = 0.04), and CD45RA+ CD69- CD294+ cell counts were higher with higher maternal urinary arsenic concentrations (*p* = 0.01). In placental samples (*N* = 70), higher *in utero* urinary arsenic concentrations were associated with the expression of IL1β (*p* = 0.03), indicating immune cell alterations with arsenic exposure [37].

### Arsenic and cancer: epidemiological evidence

Epidemiological studies from high arsenic exposure areas have elucidated that arsenic in drinking water and several cancers, including lung, bladder, liver, kidney, skin, and pancreatic cancer, are associated (See Table 1: Human organs epidemiologically linked to arsenic-mediated carcinogenesis). A 2013 review article also summarizes various human health effects by arsenic [38].

**Table 1:** Human organs epidemiologically linked to arsenic-mediated carcinogenesis

Organs	Reference
Lung	38, 40, 44, 45, 46, 47, 49, 50, 51, 52, 53, 54, 56, 57, 58, 59
Bladder	38, 39, 44, 45, 46, 52, 55, 56, 58, 59
Liver	38, 52, 55, 56, 58, 59
Pancreas	42, 48, 54
Kidney	38, 46, 51, 52, 58, 59
Skin	38, 41, 59

### The U.S. studies

Two recent case-control studies in the U.S. [39, 40] gave strong evidence that inorganic arsenic exposure is causal to a higher risk of bladder and lung cancer. New England researchers [39] reported that bladder cancer risk significantly increases with increasing drinking water intake from all sources (*p*-trend = 0.003). This trend suggested that there might be a bladder carcinogen present in the drinking water. They then explored whether this trend might be attributable to arsenic in private wells, which are prevalent in the region. The results indicated a strong association with water intake among participants who derived all of their private well water from dug wells, and among those who used dug wells before 1960, when arsenical pesticides were widely used. The researchers observed that lifetime cumulative arsenic exposure from drinking water is significantly associated with bladder cancer risk in New England.

In 10 counties in New Hampshire and Vermont, a population-based case-control study [40] of 223 patients with lung cancer was conducted. The associations between low-to-moderate (< 100 ppb) arsenic in drinking water and lung cancer were examined. The lowest toenail arsenic concentration was positively associated with a three-fold risk of squamous cell and small cell lung carcinoma (*OR* = 2.99, 95% CI [1.12, 7.99]). These results supported the possibility that specific lung cancer histological types may be associated with lower levels of arsenic exposure. The findings counter-intuitively indicated a possible tumor-suppressive role of arsenic in lung cancer development in some populations exposed to arsenic. The finding also suggested that the relationship between arsenic exposure and carcinogenesis may not be straightforward.

A recent meta-analysis of six studies focused on the relationship between arsenic in drinking water and non-melanoma skin cancer in the U.S. The study suggested that skin cancer risk is associated with consumption of tap water in some areas in the U.S. In some cases the higher risk occurred at arsenic concentrations less than 10ppb, current EPA standard [41].

Although several studies focused on the association between arsenic and cancers in lung, bladder, and skin, few reports have examined the effects of arsenic on pancreatic cancer. Recently, a spatial modeling study compared pancreatic cancer patients living within close proximity of arsenic-contaminated wells in Florida (< 1 mile) and patients living more than 3 miles away. The study identified significant likelihood to be diagnosed with a cluster of PC for the patients living closer (*OR* = 2.1, 95% CI [1.9, 2.4]) [42]. These findings show that pancreatic cancer risk may be associated with drinking water from wells contaminated by arsenic, although case-controlled studies are needed to confirm these findings. The authors proposed that the sites may be appropriate for the evaluation of pancreatic cancer risk factors and the interaction of arsenic with other co-carcinogens, such as tobacco smoke, and for targeted screening and prevention studies [42].

### Chile studies

Because of intense mining activities and volcanic activities in northern Chile [43], its drinking water is contaminated by high-level arsenic. Records of arsenic concentrations are available, with many dating back >40 years [44]. Chile is one of the driest habitable places and public water systems are largely used for drinking water [44]. These characteristics make northern Chile an ideal region in which to conduct arsenic studies. Four studies [44–47] in northern Chile explored the association between arsenic exposure *via* drinking water and lung, bladder, and kidney cancers.

Steinmaus et al. (2013) [44] examined the association between drinking arsenic-contaminated water and lung and bladder cancers in northern Chile. This study is to date the largest study with data on cancer incidence (rather than mortality) and individual data (rather than ecologic) on lifetime arsenic exposure. It is also the first study to provide clear evidence that the incidence of cancers related to arsenic is likely to remain high almost 40 years after consuming high levels of arsenic. Participants were 232 patients with lung cancer, 306 patients with bladder cancer, and 640 age- and gender-matched controls. The results indicated a dose-response relationship between arsenic exposure and increasing odds ratios for lung and bladder cancer. This work also provided evidence of an almost seven-fold increase in bladder cancer and a four-fold increase in lung cancer 35–40 years after high arsenic exposure stopped. For those exposed to the highest quartile of average arsenic concentrations (> 335 ppb) in water before 1971, the odds ratio for bladder cancer was 6.50, 95% CI [3.69, 11.43]. The corresponding lung cancer odds ratio was 4.32, 95% CI [2.60, 7.17]. Odds ratios for these two cancers were also calculated for the highest arsenic exposure period of 1958–1970. The odds ratio for bladder cancer was 6.88, 95% CI [3.84, 12.32]. The odds ratio for lung cancer was 4.35, 95% CI [2.57, 7.36].

Another study in northern Chile assessed cancer incidence after early-life arsenic exposure (*n* = 221 participants with lung cancer, *n* = 160 participants with bladder cancer). The results showed that after early-life exposure to arsenic, up to 40 years after high exposure, lung and bladder cancer incidence in adults significantly increased. For those with the highest arsenic (> 800 ppb) exposure *in utero* and as children, the odds ratio was 5.2 (95% CI [3.0, 9.0]) for lung cancer and 8.1 (95% CI [4.3, 15.2]) for bladder cancer. These results are consistent with those from other studies [45, 46].

Ferreccio et al. (2013) also investigated the effects of arsenic-contaminated drinking water on kidney cancer incidence in northern Chile [46]. In 2007–2010, they conducted a case-controlled study of 122 patients with kidney cancer. For those with the highest arsenic exposure (> 1000 ppb), the odds ratio for renal, pelvis, and ureter cancer was 11.09, 95% CI [3.60, 34.16]. Although research into the association of arsenic with kidney cancers is limited, these findings provide strong evidence of an 11-fold increase in kidney cancer due to arsenic exposure.

Another case-controlled study in northern Chile [47] investigated the relationship between lung cancer and arsenic-contaminated drinking water. This project involved 152 lung cancer patients diagnosed between 1994 and 1996. The findings revealed a clear positive trend in lung cancer odds ratios and 95% confidence intervals with increasing concentrations of arsenic in contaminated water, from 1930 through 1994. The age- and sex-adjusted odds ratio for those with the highest arsenic (200–400 ppb) exposure was 8.9, 95% CI [4.0, 19.6]. A similar trend was apparent based on the period of peak arsenic exposure in Antofagasta from 1958–1970. The odds ratio for those exposed to the highest arsenic concentration (700–999 ppb) was 7.1, 95% CI [3.4, 14.8]. The authors reported that the dose-response in both groups was consistent with supralinearity. This study provided clear evidence that drinking water with inorganic arsenic increases the risk of lung cancer.

### Spain study

There is minimal literature exploring arsenic and exocrine pancreatic cancer (EPC). However, EPC risk and the concentrations of trace elements, including arsenic, in toenails were evaluated in a case-controlled study in Eastern Spain [48]. High concentrations of arsenic (*OR* = 2.02, 95% CI [1.08, 3.78], *p*-trend = 0.009) were associated with a two-fold greater EPC risk.

### China studies

In China, two studies of the effects of arsenic on carcinogenesis were conducted [49, 50]. Among Chinese tin miners, a segment was exposed to arsenic occupationally *via* smelting and/or mining [49]. There was a significant 5-fold increase in lung cancer risk in those in the highest quartile of cumulative arsenic exposure (> 16.093 IAEM; relative risk = 4.94, 95% CI [1.95, 12.54]). A similar study [50] among Chinese tin miners in Yunnan Province examined the association between arsenic exposure and lung cancer. For those exposed to arsenic for 1–23 years, the odds ratio of lung cancer was 6.8, 95% CI [2.0, 23.7]. For those with 24–55 years of exposure, the odds ratio was 19.8, 95% CI [4.4, 88.6]. This finding suggests an almost 20-fold increase in the likelihood of lung cancer after 24–55 years of arsenic exposure. Compared with those receiving no arsenic exposure, those in the highest quarter of arsenic exposure had a relative risk of 21.1 (95% CI [6.5, 68.3]) in the bivariate analysis, and a relative risk of 22.6 (95% CI [4.8, 106.4]) in the multivariate analysis. Bivariate and multivariate analyses suggested a monotonic increase in risk with exposure. For occupational exposures, the relative risk of lung cancer for subjects exposed to arsenic from smelting alone was 12.3 (95% CI [1.7, 91.9]), while the risk from mining alone was 8.8 (95% CI [2.4, 32.2]), and the risk of smelting and mining combined was 22.0 (95% CI [4.9, 98.2]). Taylor et al. (1989) [50] observed that, among arsenic-exposed individuals, those who developed lung cancer were exposed to arsenic for a longer duration, but at a lower average intensity. This finding indicates that the duration of exposure may be more important than intensity in the development of lung cancer. The more than 20-fold increase in lung cancer incidence after arsenic exposure has important implications.

### Argentina studies

Arsenic exposure from drinking water in Argentina is well-documented. The exposure is often above 100 ppb and up to 2000 ppb, making Argentina a suitable region for arsenic exposure research [51, 52]. An ecologic study conducted in Cordoba [51] examined the relationship between drinking arsenic-contaminated water and kidney, lung, skin, bladder, and liver cancers. The authors found increasing trends for kidney and lung cancer mortality with drinking water with higher arsenic. The dose-response relationships in standardized mortality ratios (SMR) for kidney cancer, from high to low arsenic exposure, were 1.57, 1.33, and 0.87 for men, and 1.81, 1.36, and 1.00 for women (*p* < 0.001 in trend test for both). For lung cancer, the SMR from high to low arsenic exposure were 1.77, 1.54 and 0.92 for men, and 2.16, 1.34, and 1.24 for women (*p* < 0.001 in trend test for both). These results are similar to those from another study [52] that examined bladder cancer (2.14, 1.28, and 0.80 for men, 1.81, 1.39, and 1.22 for women). However, there was only a small positive trend for liver cancer. Skin cancer mortality was only elevated for women in the high exposure group, while an unexpected increase in mortality was observed in men in the low exposure group. These results add to the evidence that arsenic ingestion increases the risk of lung and kidney cancers. However, the association between arsenic and mortality from liver and skin cancers was not clear [51].

Another study of 26 Cordoba Counties [52] investigated bladder cancer mortality in 1986–1991. The authors grouped counties into high, medium, and low exposure categories. Bladder cancer SMRs were higher in the counties with arsenic exposure. The results demonstrated a significant dose-response relationship between ingested inorganic arsenic and bladder cancer. The corresponding SMRs were 2.14 (95% CI [1.78, 2.53]), 1.42 (95% CI [1.14, 1.74]), and 0.80 (95% CI [0.66, 0.96]) for men, and 1.82 (95% CI [1.19, 2.64]), 1.58 (95% CI [1.01, 2.35]), and 1.21 (95% CI [0.85, 1.64]) for women.

### Japan studies

Residents of Japan's Niigata Prefecture lived in an arsenic-polluted area and used well water containing inorganic arsenic. A Japanese historical cohort study [53] investigated the long-term effect of high exposure to ingested arsenic (≥ 1000 ppb). From 1949 until 1952, 454 residents who used well water containing inorganic arsenic were followed. The exposure period was estimated at about 5 years (1955–1959). The calculated SMR was 15.69 (95% CI [7.38, 31.02]) for lung cancer and 31.18 (95% CI [8.62, 91.75]) for urinary tract cancer. Although there is a lack of research regarding urinary tract cancer and arsenic exposure, this study supports the possibility of an increased risk of urinary tract cancer, with an estimation of a 30-fold increase in cancer incidence after high arsenic exposure. A recent study suggested a link between mortality from pancreatic cancer and childhood exposure to arsenic-contaminated milk powder [54].

### Taiwan and Bangladesh studies

Convincing evidence has also been provided by five important Taiwanese studies [55–59]. Chen and Ahsan (2004) [55] estimated excess lifetime mortality rates for the internal cancers related to arsenic in Bangladesh from a sample of 65, 876 people. Data on risk factors, such as gender, age, arsenic exposure, and cancer mortality, were obtained from a study conducted in Taiwan, since no such estimates were available in Bangladesh. Lifetime excess risks (per 100,000 population) of mortality from liver, bladder, and lung cancers attributable to drinking water with arsenic were 0.9, 21.5, and 175.9 for males and 3.4, 2.1, and 48.3 for females, respectively. Overall lifetime excess mortality risks (per 100,000) from the three cancers combined were 198.3 for males and 53.8 for females, with an average across-gender lifetime risk of 126.1. The results indicated at least a doubling of lifetime mortality risk from liver, bladder, and lung cancers (a rate of 229.6 per 100,000 vs. a rate of 103.5 per 100,000) in Bangladesh due to arsenic in drinking water.

Another study [56] presented a risk assessment for mortality from bladder, lung, and liver cancers attributed to drinking arsenic-contaminated water, based on data from 42 villages in an arsenicosis-endemic region of Taiwan. The number of deaths were extracted for 1973–1986 by age group and gender. The results depicted a strong positive correlation between arsenic exposure and bladder, lung, and liver cancers. For bladder cancer, the SMR for those in the highest arsenic exposure category (> 600 ppb) was 32.71. The corresponding SMR for lung cancer was 5.14, while the SMR for liver cancer was 2.17. For all cancers combined, the corresponding SMR was 4.86. In all 42 villages, the SMR for bladder cancer was 10.05 for males and 19.04 for females. The SMR for lung cancer was 2.20 for males, and 3.54 for females. Finally, the SMR for liver cancer was 2.06 for males and 3.68 for females. For each cancer type, the cancer risk tended to be higher in females than males. These findings strongly suggest that bladder, lung, and liver cancers are associated with arsenic exposure.

To further examine the dose-response relationship between ingested arsenic exposure and lung cancer risk, a follow-up study [57] was conducted in southwestern and northeastern Taiwan. The average follow-up time was eight years. Among 83,783 people in the study, 139 patients were newly diagnosed with lung cancer during a follow-up period. After adjustment for cigarette smoking and other risk factors, there was a monotonic trend of lung cancer risk by arsenic level in drinking water of less than 10 to 700 ppb or more (*P* = 0.001). For those with the highest arsenic exposure (≥ 700 ppb), the relative risk was 3.29 (95% CI: 1.60–6.78), which suggests a three times greater risk of lung cancer. This finding confirms that the lung cancer risk is associated with arsenic exposure in a dose-dependent manner.

Hopenhayn-Rich et al. [52, 58] examined the dose-response relationships between arsenic levels in water in Taiwan and lung, liver, bladder, and kidney cancers. The estimates of death from one of these cancers due to a lifetime consumption of water containing arsenic of 50 ppb at a rate of 1 L/day were 9.4/1000 for males and 17.3/1000 for females, with an average of 13/1000. The observed difference in male and female risk is in part due to the lower background cancer rates for women in Taiwan. Additionally, according to the EPA, men in Taiwan drink twice as much water as do women, and the difference in water consumption (arsenic exposure) also decreased the carcinogenicity estimate for men. Hopenhayn-Rich et al. (1996) [52] also estimated a similar statistic for the U.S. The authors estimated that average U.S. water intake of 1.6 L/day and an average arsenic water level of 2.5 pg/L. An estimated lifetime risk of death from liver, lung, bladder or kidney cancer due to arsenic in drinking water was extrapolated to be 1/1000. The results provide persuasive evidence that inorganic arsenic is a cause of several human cancers.

Chen et al. (1992) [59] conducted research in Taiwan to examine a similar relationship during the years 1973–1986. Among a total of 898, 806 persons in the study, there were 202 liver cancer, 304 lung cancer, 202 bladder cancer, and 64 kidney cancer deaths reported. A significant dose-response relationship was observed between arsenic level in drinking water and cancer mortality. No significant gender differences in arsenic-induced carcinogenic responses were observed. In both males and females, mortality rates increased significantly with age for all cancers. This study also revealed similarity and difference in cancer development between genders. For example, males had a higher mortality from liver cancer in almost all age groups. Males ages 50 or older had a higher mortality from lung cancer than females in the same age group. Males and females had similar age-specific mortality rates from bladder and kidney cancers. In liver, lung, bladder, and kidney, significant dose-response relationships were observed between the ingested arsenic level and mortality from cancer. The researchers reported significant increase in mortality from cancers of the liver, lung, bladder, kidney, and skin among residents.

### Use of arsenic as an anticancer reagent

As noted above, arsenic can cause cancer in humans. However, with their ROS-generating and cell-damaging, and, thus, cell-death-inducing, effects, arsenic compounds have also been used for cancer chemotherapy at higher doses. In the past 15 years, arsenic trioxide (As_2_O_3_; ATO) has been applied to acute promyelocytic leukemia (APL) that is unresponsive to “first line” agents, like all-trans retinoic acid (ATRA). The effect of ATO is attributed to its ability to specifically degrade PML/RAR alpha, a core driving oncoprotein of APL [60]. ATO showed greater treatment efficacy with considerably less hematological toxicity, when used alone or in combination with ATRA for APL [61, 62]. ATRA and anthracycline-based chemotherapy is a highly efficient and widely used strategy for APL with cure rates above 80%. However, the treatment is associated with risk of severe infections and occurrence of secondary leukemia. Overall, ATRA+ATO treatments have revolutionized the treatment of APL, resulting in cures for the majority of patients [63, 64].

With this success, application of ATO to other cancers has been considered, and is at various clinical trial stages [e.g., 65, 66]. For example, in the U.S., there are currently 166 arsenic-related clinical trials, including studies of solid tumors in skin (melanoma), liver, lung, brain, kidney, and pancreas (as of March 7, 2016) [67]. Current limitations to ATO-use include various cardiovascular issues (e.g., cardiotoxicity, myocarditis, ST segment depression, T-wave changes, QT prolongation, multifocal ventricular tachycardia, and sudden death) [68].

### Arsenic and cardiovascular issues

Arsenic exposure can lead to cardiovascular/endothelial dysfunction [69]. Some reagents have been reported to be able to ameliorate Arsenic-mediated endothelial toxicity *in vivo*. The reagents include linagliptin (a drug for type 2 diabetes) [70], a combination of telmisartan (an angiotensin receptor blocker used for hypertension) and omega 3-fatty acids [71], atorvastatin (a statin) [72], rosiglitazone (a drug for type2 diabetes) [73], and benfotiamine (a thiamine derivative) [74]. Further study is needed to address this clinically important issue of arsenic-mediated cardiovascular problems.

### Ameliorating the effects of arsenic exposure with antioxidants and other reagents

Given the ethical prohibition of experiments on human populations, rodent models are the best current approach with which to investigate cancer prevention measures. Studies of rodent models have revealed possible countermeasures against arsenic-induced damage and, thus, against carcinogenesis. Consistent with the notion that ROS generation is a major effect of arsenic, antioxidants and reagents that can enhance the oxidative stress response pathway have been shown to ameliorate the damage caused by arsenic treatment. Factors and co-factors involved in the methylation-mediated arsenic detoxification-excretion process and metal chelators have shown mitigating effects.

### [A note regarding the animal-based studies listed]

It should be noted, however, that (i) many animal-based studies mentioned here used a higher dose of arsenic (e.g., order of ppm instead of environmentally relevant ppb doses) to provoke measurable biological responses, that (ii) the endpoints of most of these studies were oxidative stress- or damage-related biomarkers, and not carcinogenesis, which requires a considerably longer time for experiments and evaluation, and that (iii) interpreting and translating results of animal-based studies to humans requires caution. To demonstrate the results' applicability and usefulness beyond reasonable doubt, we propose a measurable, biomarker-based approach both in animal models and in humans.

### Metal chelators

The first-line defense against acute arsenic poisoning is to reduce the amount of arsenic in the body. Metal chelators have been used for this purpose. However, major issues in the use of metal chelators include their lack of controls in human clinical situations, the difficulty in assessing their effects, and that chelators can increase the uptake of arsenic by the brain. Only partial success have been achieved by chelator treatments, due to the side effects and limitations (e.g., low therapeutic index, low cellular membrane penetration, toxicities to kidney and liver, and poor specificity). Further, animal-based experiments frequently employed administration routes with questionable clinical relevance, such as intramuscular injection, adding difficulty to the evaluation [75].

### Cysteine

The amino acid cysteine carries sulfhydryl groups that have high binding affinity to circulating arsenic. Supplementation with cysteine can aid in excretion of arsenic from the system. Keratin observed in hair or nails has high cysteine content. That is the reason why arsenic concentration is higher in the tissues [76].

### Dimercaptopropanol (British anti-Lewisite; BAL), meso-2, 3-dimercaptosuccinic acid (DMSA), sodium 2, 3-dimercapto-1-propanesulfonate (DMPS) [77]

BAL, DMSA, and DMPS are metal-chelating agents that have been tested for the treatment of arsenic poisoning [78]. Limited epidemiological studies have indicated that DMPS chelation alleviated the symptoms of arsenicosis in arsenic-exposed groups, but DMSA chelation did not [79, 80].

### Vitamins and trace elements

#### Vitamin C (ascorbic acid) and E (alpha-tocopherol)

Both are known anti-oxidants. In albino rats, thirty days of a high dose of 100 ppm arsenic in drinking water induced protein oxidation, DNA double-strand breaks, and DNA-protein crosslinks in blood, liver, and kidney. Concomitant supplementation of antioxidants Vitamin C (200 mg/kg, gavage once per day) and/or Vitamin E (400 mg/kg, gavage) with arsenic significantly reduced the arsenic-induced damage, both alone and in combination [81].

### Vitamin B12 and folic acid

Folate is an essential cofactor to generate methionine, which is the source of the methyl group in the methylation process during arsenic detoxification. Vitamin B12 acts as a methionine synthase enzyme to add the methyl group in the methylation process. Thus, a combination of Vitamin B12 and folic acid was hypothesized to facilitate arsenic detoxification. Folic acid is also a radical scavenger and considered an antioxidant. Arsenic-induced islet cellular toxicity was assessed in rats treated with arsenic (3 mg/kg/day for 30 days). The rats showed a significant increase in ROS indicators (i.e., malondialdehyde (MDA), nitric oxide (NO), and hydroxyl radical (OH-) formation) in the pancreatic tissue, while cellular content of antioxidant glutathione (GSH) and the activity of antioxidant enzymes (superoxide dismutase (SOD) and catalase (CAT)) were low. The serum levels of inflammatory markers (i.e., tumor necrosis factor-alpha (TNF-alpha) and IL-6) were significantly high in these animals, while the number of islet cells fell markedly. Vitamin B12 (0.63 μg/kg/day for 30 days) and folic acid (36 μg/kg/day for 30 days) reduced arsenic-induced cellular oxidative and inflammatory toxic changes [82].

### Zinc

Zinc is an antioxidant [83]. Rats received sodium arsenite in drinking water (100 ppm), zinc sulfate in drinking water (227 mg/L), or a combination for 3 months. Histological studies of the liver showed lobular inflammation, focal hepatitis, hepatocyte degeneration, and severe periportal necrosis. Administration of zinc to arsenic-treated rats significantly decreased the level of oxidation marker lipid peroxidase, but increased the levels of anti-oxidative markers (glutathione, SOD, glutathione peroxidase, glutathione reductase, and catalase activity). Histological damage was also reduced [84]. Work with a mouse model suggested that 4% zinc in water consumed during the perinatal period of pregnancy can ameliorate the possible behavioral and biochemical toxicities in the offspring [85].

### Selenium

Selenium is an essential dietary trace element and an antioxidant. Selenium closely localizes to the active site of many antioxidant enzymes (e.g., glutathione peroxidase (GPx) and thioredoxin reductase). Rats treated with sodium arsenite (13 ppm in drinking water) for 20 weeks displayed liver damage, as measured by serum ALT, AST, GLB, hepatic lipid peroxidation, activity of antioxidant enzymes (SOD, GPX, CAT), and expression of marker genes (SOD1, GPX, CAT, Txnrd1, HSP70, HO-1). Co-feeding with sodium selenite (17 ppm) improved the markers, indicating reduced hepatic damage after treatment [86].

### Methylation-mediated arsenic detoxification/excretion pathway components

#### Glutathione

Glutathione is an essential factor involved in arsenic methylation, and is an important antioxidant for ROS inhibition. Mice were treated with a high dose of arsenic (50 mg/L) for 10 days, followed by i.p. injection with 200–800 mg/kg glutathione, every 12 h for two days. Buthionine sulfoximine, a scavenger of glutathione, was also tested. Arsenic treatment increased arsenic species in the liver and decreased glutathione and antioxidant capability in the blood. Glutathione injection alleviated the effects of arsenic and increased dimethyl arsenic excretion in the urine, while buthionine sulfoximine showed opposite effects, indicating the importance of glutathione in reducing arsenic-mediated damage [87].

### S-adenosylmethionine (SAM)

Methyltransferases that use SAM as a methyl group donor catalyze detoxification process of inorganic arsenic. *In vitro* assays indicated that supplementation with SAM (170 nM) reduced the frequency of cells with micronuclei and cytoskeleton defects after treatment with sodium arsenite [88].

### Plant-derived polyphenols and other compounds with antioxidant properties

#### Grape seed extract (GSE)

Sprague-Dawley rats were exposed to arsenic in drinking water (30 ppm), with or without GSE (100 mg/kg), for 12 months. Co-treatment with GSE significantly reduced ROS, modified various biomarkers indicative of arsenic damage, and improved renal function. The authors suggested that GSE effects are mediated by the suppression of Nox and inhibition of TGF-β/Smad signaling activation [89].

### Black tea/green tea

Tea (*Camellia sinensis*)-derived catechins have demonstrated antioxidant properties and ameliorating effects on ROS-mediated damage. In Swiss albino mice administered 500 ppb sodium arsenite for 9, 15, or 22 days, both green and black (Assam, Darjeeling) tea significantly reduced oxidative DNA damage markers 8-oxoguanine DNA glycosylase (OGG1) and 8-hydroxy-2´-deoxyguanosine (8OHdG). The phytochemicals induced various DNA repair enzymes, such as PARP1, DNA β-polymerase, XRCC1, DNA ligase III, DNA protein kinase (catalytic subunit), XRCC 4, DNA ligase IV, and DNA topoisomerase Iiβ. Thus, the authors suggested that tea polyphenols may effectively counter oxidative DNA damage and inhibition of DNA repair induced by sodium arsenite [90–92].

### Green tea and Vitamin C in combination

Wistar rats were given 10 mg/kg of trivalent inorganic arsenic by gavage, 5 days/week for 6 weeks. Vitamin C solution (1 g/l) or green tea infusion (2.5 g in 500 ml boiled water) as antioxidants were given in the drinking fluid. Arsenic caused an increased latency in cortical-evoked potentials, and a decrease in tail nerve conduction velocity. The antioxidants countered the latter. There was a moderate correlation between the level of arsenic in the brain, electrophysiological changes, and lipid peroxidation. The effect of green tea was stronger compared with that of vitamin C. Green tea also diminished lipid peroxidation induced by arsenic [93].

### Resveratrol

The natural compound resveratrol has multiple phenolic hydroxyl groups, and shows a strong cytoprotective capacity against ROS. In a Chinese Cat model, resveratrol facilitated arsenic metabolism and decreased oxidative stress, thus protected against ATO-induced nephrotoxicity [94]. However, resveratrol aggravated mitochondrial damage, intracellular oxidative stress, and apoptosis in cancer cells with ATO, indicating synergistic anti-leukemia activity. Resveratrol also neutralized cardiotoxicity. The apparent difference in terms of oxidative stress response was explained as due to the difference in intracellular ROS environments between cancer cells and normal tissues [95].

### Quercetin

Quercetin is a flavonoid found in many plants and is a potent oxygen free radical scavenger. In rats, daily oral arsenic (12 mg/kg) treatment for four consecutive months induced hepatic and neuronal cell damage. Liposomal quercetin (2.71 mg QC/kg) injected s.c. into arsenic-treated rats twice a week for four months prevented the upregulation of cytochrome c expression in liver and brain [96, 97]. In testis of rats, quercetin (50 mg/kg) co-administered orally with arsenic (50 ppm in drinking water for 49 days) prevented arsenic-induced histopathological changes, including increased luminal diameter and reduced epithelial height and tubular diameter [98].

### Curcumin

Curcumin is an active ingredient of turmeric. In mice, 100-ppm arsenic induced hepatic injuries and oxidative stress. But curcumin (200 mg/kg, gavage, twice weekly for 6 weeks) attenuated the damages through multiple pathways including activation of the Nrf2 pathway, and promotion of arsenic methylation and urinary excretion [99, 100]. In a West Bengal study with chronically arsenic-exposed (95–210 ppm in water) asymptomatic human volunteers, supplementation of curcumin with piperine (a component of black pepper; 20:1) at a dose of 2 × 500 mg/day was given for 3 months. The intervention retarded ROS generation and lipid peroxidation, reduced DNA damage, and raised the level of antioxidant activity [101].

### Selenium and curcumin in combination

In pregnant BALB/c mice, drinking of 42.5 or 85 ppm arsenic in water substantially changed the counts of the enriched population of adult stem cells, progenitor cells, and differentiated cells in the epidermis of their zero-day-old neonates, indicating that arsenic suppressed stem cells in neonates. The combined intake of selenite (5.6 mg/kg) and curcumin (100 mg/kg) *in utero* prevented the disruption of homeostasis and associated biochemical changes, e.g., levels of Nrf2, NFkB, IkB, TNF-α protein products, and GSH. The authors suggested that curcumin activates Nrf2 and enhances GSH biosynthesis, and the selenium and GSH complex aids in the release of arsenic [102].

### Pomegranate

Mice were treated with arsenic (10, 50, and 100 ppb for 30 days), with or without pomegranate fruit extract. Pomegranate extract significantly reduced arsenic-induced hepatotoxicity and apoptosis by modulating the ROS/Nrf2/p53-miR-34a axis [103].

### Selenium and pomegranate in combination

Rats were treated with oral sodium arsenite (5.5 mg/kg daily), with or without daily oral administration of sodium selenite (3 mg/kg) and/or 100 mg/kg of pomegranate ethanol extract in water. After 3 weeks, authors assessed the parameters for hepatotoxicity (e.g., histopathology, ALT, AST, serum albumin, MDA, NO, IL-6, Nrf2). Significant improvements were observed in the group treated with both pomegranate extract and selenium. Multiple pathways, including inhibition of lipid peroxidation and protein oxidation, inhibition of IL-6, and restoration of antioxidant status, were proposed to prevent liver injury [104].

### Dihydroxy-isosteviol-methyl-ester from Pulsatilla nigricans

Although fresh P. nigricans plant (wind flower; native to North America, Europe and Asia) is an irritant and toxic, the dried plant has been used as a folk medicine against food poisoning and against conditions of the reproductive systems. A biologically active ingredient, dihydroxy-isosteviol methyl ester (DIME), was identified recently. Mice were treated with a sub-lethal dose of sodium arsenite (20 mg/kg/day), and the effect of an ethanol extract of P. nigricans (35 mg/kg gavage, twice daily) on testicular toxicity after 30, 60, and 90 days was examined. Arsenic-induced changes, including ROS-related biomarkers, DNA damage in testes cells, and histopathological damage to testes, were significantly reduced with P. nigricans extract. Thus, an ethanol extract of P. nigricans as an antioxidant and potent anti-apoptotic agent could provide protection or recovery to damaged cells and/or testis tissues. However, a higher dose of the extract was toxic. Therefore, further toxicological analysis should be performed [105].

### Others

#### Taurine

Taurine (2-aminoethanesulfonic acid) is a conditional amino acid and can act as a direct and indirect antioxidant. Taurine is the major free amino acid in the male reproductive system, and was shown to be cardioprotective. Using rats, the protective role of taurine against arsenic-induced testicular oxidative impairment was investigated. Oral administration of taurine (100 mg/kg for 5 days) effectively countered oxidative stress, attenuated testicular damage, and ameliorated apoptosis in testicular tissue by controlling Bcl-2/Bad, phospho-ERK1/2, phospho-p38, phospho-Akt, and NF-kappaB. Taurine also played a similar beneficial role *via* mitochondrial-dependent pathways [106].

### acetyl-L-carnitine (ALC)

ALC is an acetyl ester of the trimethylated amino acid l-carnitine (LA). ALC synthesis is especially high in the brain and liver. ALC is an effective anti-inflammatory, cytoprotective, neuroprotective, metal chelator, and anti-apoptotic agent. ALC plays a role in energy production. Acting as the “shuttle” for long-chain fatty acids, the ALC transports fatty acids between the mitochondria and the cytosol for subsequent β-oxidation. Rat were treated with arsenic (20 mg/kg for 28 days), with or without ALC (300 mg/kg). Biomarkers were assessed in plasma, kidney, liver, brain, heart, and lung. ALC demonstrated significant protective effects against arsenic [107].

### Lectin from mushroom (Pleurotus florida)

Dietary lectin from Pleurotus florida, a widely cultivated, edible oyster mushroom, was suggested to have hydroxyl radical scavenging and lipid peroxidation inhibition activities. Lectin extracted from the mushroom was tested on rats showing arsenic-induced hepatic oxidative stress. A control group and several treatment groups [arsenic (20 ppm) in drinking water, arsenic plus oral Vitamin C (25 mg/kg), and arsenic plus oral mushroom lectin (150 mg/kg for 12 weeks)] were compared. Lectin showed significant protection that was comparable to or slightly better than Vitamin C (positive control) against arsenic-induced hepatic damage, as indicated by arsenic exposure biomarkers, including activities of SOD and CAT, production of NO, and LPO level [108].

### Alpha-lipoic acid (ALA)

ALA is a fatty acid in mitochondria, is involved in energy metabolism, and can act as an antioxidant *in vitro*. Rat pups were divided into control groups receiving either no treatment or distilled water injected intraperitoneally (i.p.), and experimental groups receiving either sodium arsenite alone (1.5 and 2.0 mg/kg) or arsenite followed by ALA (70 mg/kg) i.p. from post-natal day 4–15. Retention memory was tested with a maze test, and oxidative stress markers (GSH, SOD) in the hippocampus were evaluated. Administration of ALA improved memory retention and increased the levels of hippocampal GSH and SOD [109].

### Medicinal herbs

(*Boerhavia diffusa, Corchorus olitorius [jute]*, *Salvia miltiorrhiza* [Chinese sage or Danshen in Chinese], *Silibum marianum* [milk thistle], *Terminalia arjuna* [Arjun tree]); these herbs, used in local traditional medicine systems, were listed as cardioprotective upon arsenic exposure [68].

### Biomarkers for arsenic exposure

#### In rodent models

Various studies that primarily used rodent models evaluated the ability of reagents to ameliorate the negative effects of arsenic exposure. Evaluations included: (i) histopathological evaluation of tissues of interest (e.g., blood, liver, testis, brain), (ii) DNA damage evaluation (e.g., double-strand break [γH2AX, comet assay], oxidative DNA adducts), (iii) ROS-related biomarkers and antioxidant enzymes (e.g., glutathione-S-transferase [GST], superoxide dismutase [SOD], catalase [CAT]) (iv) anti-oxidative activity, which decreases with arsenic exposure, in tissues of interest, (v) lipid oxidation as a marker of ROS exposure, (vi) markers for tissue damage, such as aspartate aminotransferase (AST), alanine aminotransferase (ALT), lactate dehydrogenase (LDH), and bilirubin, and (vii) tissue-specific cellular assay such as sperm motility. In rare cases, (viii) behavioral evaluation (e.g., maze test, locomotor activity) was used (Table 2-1: Arsenic exposure biomarkers in rodent models).

### In humans

Arsenic exposure and its effects in humans have been measured using various biochemical markers, including the direct measurement of arsenic itself in hair, urine, or toenails. However, there is no single biomarker that is both sensitive and specific enough to be applicable to the human population [110]. *In vivo* models and some population-based studies have shown promising results [111–119]; therefore, some of these biomarkers are presented in Table 2-2 (Table 2-2: Proposed arsenic exposure biomarkers in humans).

**Table 2-1:** Arsenic exposure biomarkers in rodent models

Biomarkers	Examples	Study examples
(i) histopathological evaluation of tissues of interest	observation/histopathological analysis of blood, liver, testis, brain, or kidney	82, 84, 94, 98, 103, 105, 106, 107
(ii) DNA damage evaluation	double-strand break [γH2AX, comet assay], oxidative DNA adducts	81, 88, 90, 91, 105
(iii) ROS-related biomarkers and antioxidant enzymes	glutathione-S-transferase (GST), catalase (CAT), superoxide dismutase (SOD)	82, 84, 86, 91, 100, 103, 107, 108, 109
(iv) anti-oxidative activity, which decreases with arsenic exposure, in tissues of interest	antioxidant activity in whole blood	99, 100
(v) protein or lipid oxidation as ROS exposure marker		81, 96, 98, 107, 108
(vi) markers for tissue damage	alanine aminotransferase (ALT), aspartate aminotransferase (AST), lactate dehydrogenase (LDH), and bilirubin; NADPH	89, 99, 104, 107
(vii) cellular assay	sperm motility	106
(viii) behavioral evaluation	maze test, locomotor activity	85, 109

**Table 2-2:** Proposed arsenic exposure biomarkers in humans

Proposed Biomarkers	STUDY
Testosterone	111
Guanine	
Hippurate	
Acetyl-N-formyl-5-methoxykynurenamine	
Serine	
Soluble receptor for advanced glycation end products (sRAGE)	112
Matrix metalloproteinase-9 (MMP-9)	
MMP-9/tissue inhibitor of metalloproteinase (TIMP-1)	
Clara cell protein	113
Beta2-microglobulin	
Retinol Binding Protein (RBP)	
MicroRNA (miRNA)	114
[i.e., let-7a, miR-107, miR126, miR-16, miR-17, miR-195, miR-20a, miR-20b, miR-26b, miR-454, miR-96, miR-98]	
DNA damage biomarkers:	115
8-oxo-7,8-dihydro-2′-deoxyguanosine (8-oxodG)	
N (7)-methylguanosine (N-7-MeG)	
Matrix metalloproteinase-2 (MMP-2)	116
Matrix metalloproteinase-9 (MMP-9)	
Serum thioredoxin1 (TRX1)	117
Matrix metalloproteinase-9	118
Myeloperoxidase	
Plasminogen activator inhibitor-1	
Soluble E-selectin	
Soluble intercellular adhesion molecule-1 (ICAM-1),	
Soluble vascular adhesion molecule-1 (VCAM-1)	
8-hydroxy-2′-deoxyguanosine (8-OHdG)	119
Micronuclei (Human foreskin fibroblasts-HFFs)	88
Reactive oxygen species (ROS)	95
Mitochondrial membrane potential (MMP)	
Superoxide dismutase	

### Future public health applications of the biomarkers and reagents ameliorating the effects of arsenic

If the rodent-based results can be translated to humans, the use of the reagents may be used to treat arsenic-induced diseases, including cancers. However, the blind application of these reagents should be discouraged at the current stage, because they may result in side effects and/or other unexpected negative health impacts. Hence, their use should be justified with biomarker analysis for arsenic exposure, and should be consistent with the emerging Precision Prevention approach, which is a segment of Precision Medicine. In the Precision Prevention approach, once arsenic exposure-related biomarkers in humans are validated and used for screening, this would warrant initiation of consultation and possibly the use of reagents that ameliorate the effects of arsenic. With recent technological progress, especially in systems biology and biomarkers research, the clinical application of Precision Prevention is predicted in the not-so-distant future.

### Conclusions

Environmental arsenic exposure remains a serious public health issue worldwide. This review provides an overview of the multifaceted effects of arsenic exposure. The epidemiologically associated human diseases include a variety of cancers (e.g., lung, urinary bladder, liver, kidney, pancreas, skin) and cardiovascular disease. Major biological effects that may link arsenic to the diseases include the generation of ROS leading to oxidative stress and DNA damage, induction of epigenetic DNA modification, induction of genomic instability, inflammation and immunomodulation. With rodent models, reagents, mainly antioxidants, have been tested to determine whether these agents can ameliorate the health effects of arsenic. Although few have been tested on humans in a clinical setting, the number of reagents should increase, with hopes of translational applications to prevent arsenic exposure-related diseases. Since a segment of the arsenic-exposed population may respond differently to arsenic, we propose the necessity for a biomarker-based screening program and Precision Prevention approach for individuals with high health risk and arsenic exposure.

### Search and citation criteria

References for this review were identified through PubMed searches with the terms “arsenic”, “cancer”, “prevention”, “amelioration”, and “chemoprevention” from 1990 until March, 2017. Only papers published in English were reviewed. Citation priorities were placed on chemicals/reagents confirmed with multiple studies, and on studies *in vivo* with humans and/or rodents. In the case of multiple publications on the same reagent, the most recent or an exemplar publication was chosen. The final reference list was generated based on originality and relevance to the scope of this review.
